# Postoperative recovery and its association with health-related quality of life among day surgery patients

**DOI:** 10.1186/1472-6955-11-24

**Published:** 2012-11-13

**Authors:** Katarina Berg, Karin Kjellgren, Mitra Unosson, Kristofer Årestedt

**Affiliations:** 1 Division of Nursing Science/Department of Medical and Health Sciences, Faculty of Health Sciences, SE-581 85, Linköping, Sweden; 2 Department of Social and Welfare Studies, Faculty of Health Sciences, Linköping University, Norrköping, Sweden; 3 School of Health and Caring Sciences, Linnaeus University, Kalmar, Sweden

**Keywords:** Ambulatory surgical procedures, Nursing, Quality of life, Recovery of function

## Abstract

**Background:**

Day surgery holds advantages for both the patient and the health care organization. However, recovery beyond the first postoperative week and following different types of surgery has not been explored to any greater degree. The current aims were to prospectively describe postoperative recovery and health-related quality of life among different groups of day surgery patients and to explore the association between postoperative recovery and health-related quality of life 30 days after discharge.

**Methods:**

A consecutive sample of 607 adult day surgery patients undergoing orthopaedic, gynaecological or general surgery was included. Postoperative recovery was assessed on days 1, 7 and 14 using the Swedish Post-discharge Surgery Recovery scale and the Quality of Recovery-23 scale. The EQ-5D was used to assess health-related quality of life preoperatively and 30 days following discharge. A repeated measure ANOVA was conducted to evaluate postoperative recovery from day 1 to day 14 and between different surgical groups. Hierarchical multiple linear regression models were used to explore the association between postoperative recovery and health-related quality of life.

**Results:**

Postoperative recovery improved from day 1 to 14 in all surgical groups (p<0.001). The orthopaedic patients had lower postoperative recovery on day 14 compared to the general and the gynaecological patients (p<0.001). Health-related quality of life was lower among orthopaedic patients (p<0.001), even if significant improvements over time were seen in all groups. Recovery on day 7 was associated with health-related quality of life 30 days after the day surgery (p<0.05).

**Conclusion:**

Particularly orthopaedic day surgical patients seem to favour a closer follow-up in order to support recovery and thereby also positively influence health-related quality of life.

## Background

New anaesthetic and surgical technologies, along with economic and political initiatives, have led to an increase in the number of surgical procedures performed as day surgery [1]. Day surgery implies a short period of surveillance at the surgery unit before home discharge. After leaving the surgery unit, patients have to manage postoperative problems on their own together with their personal network [2,3]. Despite this, day surgery is a preferred and desired choice for most patients as it offers an efficient service with minimal disruption of personal habits and routines [4,5]. Patients want the postoperative period to pass quickly, and might be unprepared and surprised if recovery takes longer than expected [6]. However, postoperative recovery is individual and entails a composite of different physical and psychological issues. Recovery is influenced by age, gender [7], type of surgery [8,9] and social factors [4]. In Sweden, the patient’s qualification for day surgery is mainly based on the American Society of Anesthesiologists’ (ASA) physical classification, type of anaesthesia and patient’s Body Mass Index. It is the surgeon who makes the first assessment of the patient’s suitability [10]. The ASA classification consists of five classes ranging from I (a completely healthy patient) to V (a moribund patient in need of lifesaving surgery) [11]. Due to its nature, classes IV and V are not relevant to day surgery. Following day surgery, postoperative recovery is described as the patient’s perception of a return to his/her usual self [12] and as an improvement in functional status [13]. Postoperative recovery is also suggested to be an energy-requiring process, as one is returning to the preoperative level of normality and wholeness regarding physical, psychological, social and habitual functions [14]. Postoperative recovery is a solid outcome measure following day surgery [15], but recovery beyond the first postoperative week and following different types of surgery have not been explored in depth [8].

Patient-reported outcome measures (PROMs) are increasingly used to obtain patient-centred data following medical treatment and intervention. The PROMs seek to measure health-related quality of life (HRQoL) from the patient’s perspective, and have become an alternative to conventional outcome measures like mortality and morbidity [16]. HRQoL is an important indicator of surgical and medical treatments, and is particularly useful following day surgery since mortality and major morbidity are very rare events [17] and thus inappropriate to use as outcome measures. No uniform definition of HRQoL exists, but it is considered a composite of biological function, symptoms, functional status, subjective health and wellbeing [18]. Patients in day surgery have varying degrees of preoperative HRQoL, and in connection with the multifaceted concept of postoperative recovery it is important to identify factors affecting HRQoL [19]. Such studies are rare, although sleep problems, pain [20] and impaired mobility [19] have been found to be associated with reduced HRQoL following day surgery.

Over the past decades, a shift has occurred in the type of patients approved for day surgery. Previously, only healthy patients, scheduled for simple surgical procedures were approved, whereas today those with comorbidity and certain social situations, as well as those needing more complex surgical procedures, are approved [21]. Due to this changed patient selection, the holistic nature of postoperative recovery and its potential impact on day surgery patients’ HRQoL, the aims of this study were to prospectively describe postoperative recovery and HRQoL among different groups of day surgery patients and to explore the association between postoperative recovery and HRQoL 30 days after discharge.

## Methods

This prospective study was performed using self-rated questionnaires. Day surgery was defined as surgery performed on a patient who was admitted, operated on and discharged on the same day without an overnight stay at the surgery unit.

The study followed common ethical principles for clinical research. All patients were informed about the confidentiality and voluntariness of the study. The study was approved by the Regional Ethical Review Board in Linköping, Sweden (Study code 03–333).

### Patients

Patients scheduled for a day surgical procedure, aged 18 years or older and able to understand and speak Swedish, qualified for participation. Eight-hundred and fifty-one consecutive patients were eligible to be asked to participate. Of these, 76 (9%) missed being asked and 135 (16%) declined participation, resulting in 640 patients giving informed consent. Patients were recruited consecutively from a county hospital (n=100), a private surgery unit (n=270) and a day surgery unit at a university hospital (n=270) during three periods from October 2003 to January 2005. Thirty-three patients were excluded, mainly because of postoperative hospitalization, resulting in 607 patients (304 women, 303 men) ultimately being included in the study. Of the included patients, 147 (24%) did not remain in the study until its conclusion 30 days after the surgical procedure. The patients leaving the study were younger (41.3 ±14.1 years) than the remaining patients (52.4 ±15.1 years, p<0.001) and were more frequently men (n=89, p=0.003). In contrast, no significant difference was noted regarding age or gender among participating patients and those who declined participation or who missed being asked. The proportion of general surgery patients was the largest, 36% (p<0.001), among those who did not participate in the study (n=211).

### Data collection

Postoperative recovery was assessed using the Swedish Post-discharge Surgery Recovery (S-PSR) [22] and the Quality of Recovery-23 (QoR-23) [23] scales. HRQoL was assessed using the EQ-5D (three levels) [24]. Home readiness was assessed before home discharge using the Postanesthesia Recovery Score for Ambulatory Patients (PARSAP) [25]. To obtain demographic data (age, gender, residence, employment and education) and the ASA classification, a structured questionnaire and patient records were used. The patient records were reviewed manually.

The S-PSR scale is a self-rating instrument for the assessment of perceived at-home postoperative recovery following day surgery represented in a twelve-item ten-point (1–10) semantic differential scale. A recovery score is computed by dividing the patient’s individual sum score by the total possible score and multiplying the result by 100. The final range score is thus 10 to 100, with higher scores indicating positive postoperative recovery [22]. The S-PSR scale has been modified from its original [12] and evaluated in the same sample as in the present study [22]. The instrument shows satisfactory validity, reliability, data quality and responsiveness [12,22]. Internal consistency, measured with the Cronbach’s α coefficient, was 0.90 in the present study. For a baseline score, eight items possible to assess before surgery from the S-PSR scale were used: the patient’s alertness, pain, tiredness, activity, need for a daytime nap, mobility, living situation and physical exercise. These were also computed by dividing the individual score by the total possible score and multiplying by 100. The possible range for the baseline score is thus 10 to 100 as well.

The QoR-23 is a self-rating instrument with 23 items rated on a five-point scale (1–5) [23]. The items are summed with a maximum total score of 115, and higher scores indicate higher quality of recovery. The instrument can also be analysed on its three-dimensional level: physical comfort (10 items), emotional state (8 items) and physical independence (5 items). In this study an overall level of postoperative recovery was in demand, so the total score was used. The QoR-23 was modified from its original [26]. Previous evaluations in this sample support the QoR-23 for use in day surgery patients [23]. The Cronbach’s α coefficient was 0.88 in this study.

The EQ-5D (three levels) is a generic, dimensional and standardized instrument designed to measure HRQoL, and consists of a descriptive system and a visual analogue scale (EQ-VAS). The descriptive system comprises five dimensions: mobility, self-care, usual activities, pain/discomfort and anxiety/depression. Each dimension has three answer alternatives, numbered 1 to 3 when analysing: no problems (1), some problems (2) and severe problems (3) [24]. The descriptive system is able to identify 243 unique health states. A score can be assigned to each of these health states, resulting in a value of maximum 1 representing perfect HRQoL and a minimum value of −0.595 representing worst possible HRQoL (the EQ index) [27]. The EQ-VAS is a 20 cm vertical, visual analogue scale anchored on 100 (best imaginable health) and 0 (worst imaginable health) [24]. The EQ-5D has satisfying validity and reliability, and is frequently used in clinical observational studies [28,29].

The PARSAP is a ten-item instrument regarding clinical conditions following day surgery and anaesthesia, for example circulation, respiration, consciousness, dressing and pain. The variables are graded from 0 to 2, and give a maximum score of 20. A score of ≥ 18 indicates recovery sufficient for home discharge [25].

### Procedures

On arrival at the surgery unit, each patient received verbal and written information about the research project, and informed consent to participate was obtained. Demographic data, EQ-5D, ASA classification and patients’ baseline score from the S-PSR scale were collected before surgery. When the patient was discharged from the day surgery unit, home readiness was assessed and the patient received a questionnaire including the S-PSR and QoR-23 scales, along with a postage-paid envelope, to be answered at home on the first postoperative day. Identical questionnaires were sent to the patients’ homes to be answered on days 7 and 14. On day 30 the EQ-5D, along with a postage-paid envelope, was sent to the patients. Due to the requested days for assessment, no reminders were sent.

### Statistical analyses

Descriptive statistics were used to describe the sample and score distributions of postoperative recovery and HRQoL. The chi square test and a one-way ANOVA, respectively, were used when categorical and continuous data were analysed.

A repeated measure ANOVA was conducted to evaluate whether there were significant changes in S-PSR and QoR-23 scores from day 1 to day 14, and to test for differences between different day surgical groups. The ANOVA models included a between-subject variable (surgical group) and a within-subject variable (repeated measure of postoperative recovery assessed using the S-PSR or QoR-23 scale, and HRQoL assessed using the EQ index and EQ-VAS). The models also included an interaction term (Time x Group). When post hoc analyses were performed to identify differences between the three time points (days 1, 7, 14), the Bonferroni correction was used to adjust for multiple comparisons. The models were controlled for sphericity. If this condition was violated, the Greenhouse-Geisser correction was used. Since the three surgical groups differed in preoperative S-PSR scores, a one-way ANOVA was used to test for mean changes in recovery from day 1 to day 7 as well as from day 1 to day 14 between the surgical groups.

Hierarchical multiple linear regression models were constructed to explore the association between postoperative recovery and HRQoL. In the models, the EQ-VAS or EQ index 30 days following the day surgical procedure was used as outcome variables. In a first block (Model I), the S-PSR and QoR scores on day 7 were included as predictor variables. Preoperative EQ-VAS or EQ index was added as a covariate in a second block (Model II). To adjust for other known covariates, education, residence, ASA classification, type of surgery, age and gender were included in the third block (Model III). S-PSR, QoR-23, EQ-VAS, EQ index and age were treated as continuous variables. Gender (female), residence (cohabitant) and education (degree from university) were treated as dummy variables. Type of surgery and ASA classification were also treated as dummy variables, with orthopaedic surgery and ASA class 1 used as reference categories. Multicollinearity was assessed using the tolerance and variance inflation factor (VIF). The tolerance varies between 0 and 1, and a value of 1 means totally uncorrelated included variables. It is preferable that the VIF have a value of less than 2 [30]. The mean tolerance and the mean VIF were 0.80 and 1.3, respectively. The adjusted R^2^ change was used to illustrate model development.

No imputation was made for missing data, which implies that sample size slightly varies in different analyses. The level of statistical significance was set at p<0.05. Statistical analyses were conducted using SPSS 19.0 (SPSS Inc., Chicago, IL).

## Results

### Preoperative characteristics of the sample

Three-hundred and fifty-eight patients underwent orthopaedic surgery, for example arthroscopic procedure or operation for carpal tunnel syndrome. General surgery, for example operation for inguinal hernia or varicose vein surgery was performed on 182 patients, and 67 patients underwent gynaecological surgery, for instance abrasio or prolaps surgery.

No difference in age was found between the patients in the different surgical groups. The majority of the patients were classified in ASA class 1, but a group difference was identified; the general surgery patients had lower preoperative physical status compared to the orthopaedic and gynaecological patients (p=0.007). The proportion of regional anaesthesia was 21% among the orthopaedic patients (p<0.001). Seventy-six percent of all patients were cohabitating and 68% were employed, although more gynaecological patients were retired (31%) compared to the orthopaedic and general surgery patients (p=0.021). Education up to secondary school level was the most common (69%) (Table 1). Before surgery, the orthopaedic patients had a significantly lower S-PSR score, EQ-VAS and EQ index compared to the general and gynaecological patients (Table 2).

**Table 1:** Patient characteristics in the different surgical groups

	**Orthopaedic patients n=358**	**General surgery patients n=182**	**Gynaecological patients n=67**	**p-value**
Gender male/female n (%)	189/169 (53)/(47)	114/68 (63)/(37)	0/67 (100)	n/a
Age mean (SD)	49.0 (15.6)	49.9 (15.4)	52.8 (15.9)	0.194^a^
Type of anaesthesia n (%)				<0.001^b^
General	196 (55)	132 (73)	39 (58)	
Regional	75 (21)	1 (−)	1 (1)	
Local	82 (23)	21 (12)	22 (33)	
Sedation	2 (1)	14 (8)	5 (7)	
Missing	3 (1)	14 (8)	-	
ASA classification n (%)				0.007^b^
1	268 (75)	120 (66)	53 (79)	
2	78 (22)	61 (34)	12 (18)	
3	11 (3)	1	2 (3)	
Missing	1	-	-	
Residence n (%)				0.718^b^
Cohabitating	268 (75)	137 (75)	54 (81)	
Single	84 (23)	43 (24)	13 (19)	
Missing	6 (2)	2 (1)	-	
Employment n (%)				0.021^b^
Working	242 (68)	130 (71)	39 (58)	
Retired	72 (20)	41 (23)	21 (31)	
Unemployed	16 (4)	5 (3)	-	
Other	21 (6)	3 (2)	6 (9)	
Missing	7 (2)	3 (2)	1 (1)	
Education n (%)				0.582^b^
Compulsory school	103 (29)	51 (28)	19 (28)	
Secondary school	151 (42)	74 (41)	23 (34)	
Degree from university	97 (28)	51 (28)	25 (37)	
Missing	7 (2)	6 (3)	-	

^a^ One-way ANOVA.

^b^ chi^2^.

ASA = American Society of Anesthesiologists.

**Table 2:** Preoperative data from the Swedish Post-discharge Surgical Recovery scale (baseline items), EQ-VAS and EQ index from different day surgery patients (mean, standard deviation)

	**Orthopaedic patients (n=353)**	**General surgery patients (n=182)**	**Gynaecological patients (n=67)**	**p-value**		**Posthoc^a^**	
S-PSR score	68.8 (16.8)	74.7 (17.6)	77.0 (16.5)	<0.001	A	B	-
EQ-VAS	71.2 (19.6)	75.8 (16.7)	77.4 (15.1)	0.003	A	B	-
EQ index	0.658 (.260)	0.766 (.208)	0.780 (.172)	<0.001	A	B	-

^a^Significant Bonferroni posthoc tests represented by: A = orthopaedic - gynaecological patients, B = orthopaedic - general surgery patients, C = gynaecological - general surgery patients.

### Postoperative recovery

All except 32 patients were discharged with a PARSAP score ≥ 18 (m=19.1 ±0.9). No difference existed between the surgical groups. Postoperative recovery, reflected by individual items on the S-PSR scale and in the dimensions of the QoR-23 scale at day 7, is shown in Table 3. For the S-PSR, differences between the surgical groups were shown for the items pain, usual activity, mobility, expectations and normal life. Except for expectations, the orthopaedic patients scored significantly lower than the general and the gynaecological patients. The orthopaedic patients also scored significantly lower on recovery compared to the general surgery patients in the physical dependence dimension on the QoR-23 scale.

**Table 3:** Items in the Swedish Post-discharge Surgery Recovery (S-PSR) and dimensions in the Quality of Recovery-23 (QoR-23) scales in all patients and in different day surgical groups on postoperative day 7 (mean, standard deviation)

		**All patients (n=471)**	**Orthopaedic patients (n=278)**	**General surgery patients (n=137)**	**Gynaecological patients (n=56)**	**p-value**		**Posthoc^b^**	
S-PSR items (score 1–10)^a^									
1.	Alertness	7.4 (2.3)	7.3 (2.3)	7.4 (2.3)	7.5 (2.5)	0.696	-	-	-
2.	Pain	7.1 (2.3)	6.7 (2.4)	7.4 (2.2)	8.4 (2.0)	<0.001	A	B	C
3.	Tiredness	7.0 (2.1)	7.0 (2.1)	6.8 (2.2)	7.1 (2.2)	0.548	-	-	-
4.	Usual activity	6.3 (2.6)	5.7 (2.6)	7.0 (2.5)	7.2 (2.6)	<0.001	A	B	-
5.	Daytime nap	7.3 (2.6)	7.3 (2.6)	7.2 (2.6)	7.4 (2.7)	0.921	-	-	-
6.	Mobility	6.0 (2.6)	5.5 (2.5)	6.5 (2.5)	7.4 (2.4)	<0.001	A	B	-
7.	Stay at home	8.0 (2.4)	7.9 (2.4)	8.0 (2.3)	8.2 (2.6)	0.512	-	-	-
8.	Physical exercise	6.9 (2.5)	6.7 (2.5)	7.2 (2.4)	7.0 (2.7)	0.198	-	-	-
9.	Expectations	7.3 (2.5)	7.3 (2.5)	7.1 (2.5)	8.1 (2.5)	0.039	-	-	C
10.	Recovery	7.1 (2.4)	6.9 (2.4)	7.2 (2.4)	7.8 (2.3)	0.054	-	-	-
11.	Normal life	6.2 (3.0)	5.7 (3.0)	6.7 (3.0)	7.3 (3.0)	<0.001	A	B	-
12.	Frame of mind	8.1 (2.6)	8.0 (2.6)	8.1 (2.6)	8.4 (2.6)	0.628	-	-	-
QoR-23 dimensions (score 1–5)^c^									
Physical comfort (10 items, range 10–50)		45.7 (5.0)	45.4 (5.3)	46.1 (4.2)	46.2 (5.2)	0.315	-	-	-
Emotional state (8 items, range 8–40)		35.0 (5.4)	34.7 (5.8)	35.3 (4.7)	36.0 (4.7)	0.196	-	-	-
Physical independence (5 items, range 5–25)		22.4 (3.1)	21.9 (3.2)	23.4 (1.7)	22.4 (4.7)	<0.001	-	B	-

^a^ Higher score represents positive recovery.

^b^Significant Bonferroni posthoc tests represented by: A = orthopaedic - gynaecological patients, B = orthopaedic - general surgery patients, C = gynaecological - general surgery patients.

^c^Higher score represents better postoperative quality.

**Figure 1:**
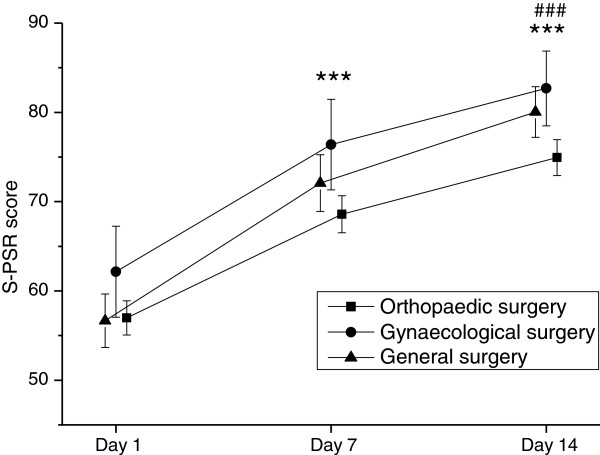
**Improvements in recovery between postoperative days 1 to 7,7 to 14 and 1 to 14 in patients undergoing orthopaedic,general and gynaecological day surgery when assessed using the Swedish post**-**discharge surgery recovery(S-PSR)scale.** Means and 95% confidence intervals. *** denotes p<0.001 compared to day 1 and ### denotes p<0.001 compared to day 7 for all three groups separately.

**Figure 2:**
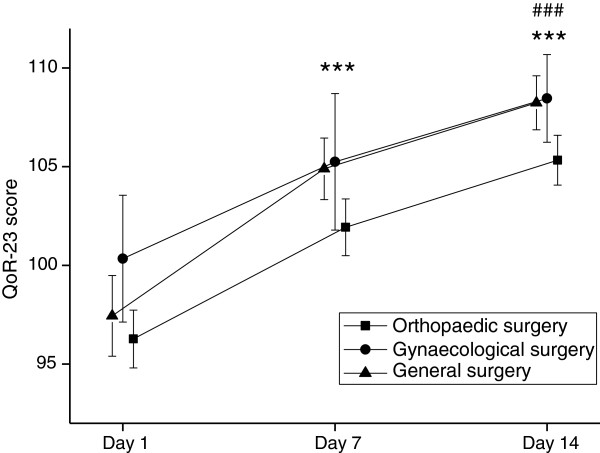
**Improvements in recovery between postoperative days 1 to 7,7 to 14 and 1 to 14 in patients undergoing orthopaedic,general and gynaecological day surgery when assessed using the Quality of recovery-23 (QoR-23) scale.** Means and 95% confidence intervals. *** denotes p<0.001 compared to day 1 and ### denotes p<0.001 compared to day 7 for all three groups separately.

Mean scores for postoperative recovery at the three time points (day 1, 7 and 14) are presented in Table 4 and Figures 1 and 2. A main effect of time (i.e. changes over the three time points) was shown for the mean S-PSR score, which increased significantly from 58 on day 1 to 78 on day 14 for all patients as a group. Post hoc analyses for time showed significant improvements at a level of p<0.001 between all time points for measurement (days 1–7, 7–14 and 1–14). A significant main effect of groups (i.e. differences between surgical groups) was demonstrated as well (Table 4). The orthopaedic patients had a significantly lower postoperative recovery score than the gynaecological patients on days 7 and 14 (Figure 1). No interaction effect was shown (Table 4). When mean changes in scores were compared between the surgical groups, a significant difference was shown initially at days 1–7 (F(2, 442)=3.03, p=0.049). This difference disappeared following the post hoc analysis. No difference was found at days 1–14 (F(2, 433)=2.52, p=0.082).

**Table 4:** Postoperative recovery over time and between different day surgical groups, based on a two-way repeated measure ANOVA (n=404)

**Scales**	**Day 1 mean (SD)**	**Day 7 mean (SD)**	**Day 14 mean (SD)**	**Source^a^**	**p-value**
S-PSR total	58.1 (17.4)	71.1 (18.0)	78.0 (16.6)	Time	<0.001
Orthopaedic patients	57.0 (16.7)	68.8 (17.5)	75.6 (16.6)	Group	0.001
General surgery patients	57.8 (18.3)	72.4 (18.6)	80.1 (16.9)	Time x Group	0.162
Gynaecological patients	64.3 (17.6)	79.7 (16.0)	84.8 (13.2)		
QoR-23 total	97.3 (12.4)	103.2 (10.8)	106.6 (9.7)	Time	<0.001
Orthopaedic patients	96.3 (12.4)	101.9 (11.1)	105.3 (10.5)	Group	0.006
General surgery patients	97.4 (12.6)	104.9 (9.0)	108.4 (8.0)	Time x Group	0.108
Gynaecological patients	102.0 (10.6)	105.9 (12.5)	108.9 (8.2)		

^a^ Time – main effect within subjects over time, Group – main effect between orthopaedic, general surgery and gynaecological day surgery patients, Time x Group – interaction effect for time and group.

S-PSR (possible score range: min 10 – max 100).

QoR-23 (possible score range: min 23 – max 115).

A main effect of time was also shown for the QoR-23 score, the mean values of which increased significantly from 97 on day 1 to 107 on day 14 for all patients as a group. Post hoc analysis for time showed significant improvements between all time points for measurements (p<0.001). A significant main effect of groups was shown (Table 4). The recovery score for orthopaedic patients was lower on day 14 compared with the general surgery patients (Figure 2). No interaction effect was shown (Table 4). Comparisons of mean changes in scores between the surgical groups showed no significant differences on days 1–7 (F(2, 425)=1.82, p=0.164) or days 1–14 (F(2, 425)=1.70, p=0.183).

### Health-related quality of life

In general, a significant main effect of time (i.e., changes from preoperative to postoperative assessment) was shown for the EQ index and EQ-VAS. Both scales increased from baseline to 30 days after surgery. The mean values of the EQ index increased from 0.727 to 0.795, and those of the EQ-VAS from 74.8 to 82.5. A significant main effect of groups was demonstrated; orthopaedic patients scored the lowest HRQoL on both the EQ index and EQ-VAS. No interaction effects were shown. The orthopaedic patients had significantly lower HRQoL scores at baseline, and despite an increase in scores they did not reach the same levels as the general and the gynaecological patients did (Table 5).

**Table 5:** Health-related quality of life over time and between different day surgical groups, based on a two-way repeated measure ANOVA

**Scales**	**Baseline (preoperative) mean (SD)**	**Day 30 (postoperative) mean (SD)**	**Source^a^**	**p-value**
EQ index (n=454)	0.727 (0.227)	0.795 (0.220)	Time	<0.001
Orthopaedic patients	0.683 (0.246)	0.744 (0.215)	Group	<0.001
General surgery patients	0.783 (0.187)	0.868 (0.203)	Time x Group	0.560
Gynaecological patients	0.796 (0.171)	0.854 (0.221)		
EQ-VAS (n=443)	74.8 (17.2)	82.5 (16.8)	Time	<0.001
Orthopaedic patients	72.6 (18.5)	80.0 (16.5)	Group	<0.001
General surgery patients	77.4 (15.0)	85.4 (17.3)	Time x Group	0.706
Gynaecological patients	78.2 (14.7)	87.5 (14.6)		

^a^ Time – main effect within subjects over time, Group – main effect between orthopaedic, general surgery and gynaecological day surgery patients, Time x Group – interaction effect for time and group.

### The association between postoperative recovery and HRQoL

The results show that both the S-PSR and the QoR-23 scores on day 7 were associated with the patients’ HRQoL 30 days after the surgical procedure (Table 6). The S-PSR and QoR-23 scores on day 7 were significantly associated with the EQ-VAS on day 30. This association was also significant in Model II when EQ-VAS before surgery was included as an adjusting covariate. Including EQ-VAS before surgery increased the explained variance by 12%. Female gender and ASA class 2 were also significantly associated with the EQ-VAS on day 30 (Model III), but the model only improved by 1%.

**Table 6:** **Hierarchical regression models for the association between postoperative recovery and health**-**related quality of life 30 days after the surgical procedure**

**EQ-VAS Day 30**	**Model I**	**Model II**	**Model III**
**n=381**	**B (SE)**	**B (SE)**	**B (SE)**
S-PSR day 7	0.390 (0.056)^***^	0.305 (0.051)^***^	0.302 (0.052)^***^
QoR day 7	0.360 (0.093)^***^	0.220 (0.086)^*^	0.244 (0.087)^**^
EQ-VAS preop		0.377 (0.042)^***^	0.368 (0.043)^***^
Degree from university			−2.099 (1.421)
General surgery			2.099 (1.501)
Gynaecological surgery			1.954 (2.279)
Age			−0.030 (0.048)
Female gender			2.837 (1.422)^*^
Cohabitant			−1.267 (1.603)
ASA 2			−3.873 (1.633)^*^
ASA 3			−3.874 (5.989)
Adjusted R^2^	0.325	0.441	0.452
Adjusted R^2^ change		0.116	0.011
**EQ-index Day 30**	**Model I**	**Model II**	**Model III**
**n**=**386**	**B (SE)**	**B (SE)**	**B (SE)**
S-PSR day 7	0.005 (0.001)^***^	0.004 (0.001)^***^	0.004 (0.001)^***^
QoR day 7	0.005 (0.001)^***^	0.003 (0.001)^*^	0.003 (0.001)^*^
EQ index preop		0.288 (0.043)^***^	0.270 (0.044)^***^
Degree from university			0.002 (0.019)
General surgery			0.068 (0.020)^**^
Gynaecological surgery			0.025 (0.030)
Age			0.001 (0.001)
Female gender			0.044 (0.019)^*^
Cohabitant			−0.002 (0.021)
ASA 2			0.001 (0.021)
ASA 3			−0.004 (0.080)
Adjusted R^2^	0.333	0.401	0.417
Adjusted R^2^ change		0.068	0.016

*=p<0.05, **=p<0.01, ***p<0.001.

S-PSR = Swedish post-discharge surgery recovery score.

QoR = Quality of recovery score.

ASA = American Society of Anesthesiologists.

The same pattern was found when the EQ index 30 days after surgical procedure was used as an outcome variable. The S-PSR and the QoR-23 scores on day 7 had a similar association as the EQ-VAS with the EQ index at day 30, and the model was explained to 33%. When the model was adjusted with the EQ index before surgery (Model II), the S-PSR and QoR-23 scores still were significantly associated with the EQ index on day 30 and the explained variance increased by 7%. Female gender was significantly associated with the EQ index on day 30 as well (Model III), as was general surgery. However, the change in explained variance was minor (1.6%).

## Discussion

In this study, all groups of patients significantly improved in recovery during the first two weeks following the day surgical procedure. However, the orthopaedic patients did not recover to the same extent as the general and gynaecological patients did. An additional finding was that recovery on postoperative day 7 was associated with patients’ HRQoL 30 days following the day surgical procedure. Also, orthopaedic patients had lower HRQoL already before surgery and did not reach the same level 30 days postoperatively as the general and gynaecological patients did.

The finding that orthopaedic patients had the lowest preoperative health condition, represented by the S-PSR baseline score, can mirror that several orthopaedic patients have preoperative discomforts like pain and impaired mobility [19,31], which in turn may affect the recovery time course. For instance, arthroscopic patients showed a slower recovery process when such preoperative discomforts were considered [8]. Also, a patient’s preoperative expectations on recovery may matter [12]. Experienced pain and ambulation difficulties before surgery can figure into positive or negative postoperative expectations. Relief from longstanding pain through surgery carries positive expectations. On the other hand there can be worries of a cumbersome rehabilitation process. In the present study, the orthopaedic patients reported more pain and reduced mobility on day 7 compared with the other groups of patients. Postoperative symptoms and discomforts are found to have an impact on recovery and activities in daily living [32]. Orthopaedic surgery is painful [9] and frequently has an effect on mobility [8], which often results in a protracted recovery period. Our focus was on recovery from a general perspective; thus only some symptoms were analysed on day 7. However, commonly occurring symptoms and discomforts such as pain, mobility, tiredness, drowsiness, sleep and nausea are included in the S-PSR and QoR-23 scales and are thereby regarded in the overall picture.

All groups of patients improved in recovery from the first postoperative day to day 14. However, the orthopaedic patients had lower recovery compared with the others. The orthopaedic patients had a lower starting position, and since no difference in mean changes in scores existed, this group of patients needed more time to reach the same level of recovery as that of the general and gynaecological patients. Many factors influence recovery following day surgery. Patient characteristics (e.g. age, body mass index, comorbidity), type of surgery, type of anaesthesia and social circumstances are such factors [21]. In this study, no difference in age existed. Comorbidity may be regarded in the light of the ASA classification in our sample. Patients in the general surgery group represented a larger proportion of ASA 2 classified patients. Despite this, the orthopaedic patients experienced lower recovery. Regarding type of surgery, arthroscopic (knee and shoulder) patients have previously been reported to experience a protracted recovery period [8,33]. General anaesthesia is a postoperative risk following day surgery, but its importance as a risk factor ought to be interpreted with caution [34]. In the present sample, the use of general anaesthesia was proportionally low among the orthopaedic patients and thus does not appear to have influenced the lower level of recovery in this group of patients; neither do social factors appear to have done. Thus, identifying patients at postoperative risk in advance seems beneficial. The identification work can be done by nurses through preoperative screening or a pre-admission appointment, and the support may consist of preoperative education [35] or a close postoperative follow-up at which the management of pain or other clinical management and self-care [2] are advised.

The orthopaedic patients had lower HRQoL than the general and the gynaecological patients both before and one month following surgery, which is in concordance with Brattwall et al. [19]. In contrast, Suhonen et al. [31] found that patients perceived HRQoL as high before as well as after the surgical procedure. These divergent results might be due to slightly different samples, even though the majority of the patients had undergone orthopaedic surgery. To increase the knowledge about HRQoL following orthopaedic day surgery, further research is needed.

The results in this study indicate that orthopaedic patients are a vulnerable group in day surgery, who may benefit from a closer contact with the health care following discharge. Increased support may facilitate overall recovery, which in turn may have a positive effect on the patients’ HRQoL. To better prepare patients for their recovery preoperative information, screening of patients and information at discharge are suggested to be priority interventions for day surgery nurses [10]. Recovery differs within the group [33] which ought to be considered when post-discharge care is planned. Validated and user friendly questionnaires, used in a telephone follow-up, could be suitable to identify patients in need of more attention.

Postoperative recovery was significantly associated with HRQoL, and to our knowledge no previous study has examined this association. When further adjusted for known covariates, ASA class 2, female gender and general surgery were associated with HRQoL. However, even if these covariates were significantly associated with HRQoL 30 days postoperatively, its contribution to the model explanation was minor and did not have any appreciable impact on HRQoL. It seems that the patients’ recovery was still the most important for perceived HRQoL following day surgery. These results indicate that the assessment of recovery one week postoperatively can be used to identify patients with risk of impaired HRQoL one month following their day surgical procedure. An additional advantage is that HRQoL has long been regarded a major predicting factor of patient satisfaction with outcomes of medical services [36]; in the day surgery context HRQoL might be considered from this perspective as well.

This study contains a number of methodological weaknesses. The sample size was large and consecutively collected; nevertheless, the gynaecological patients were few in number. No sample size calculation was performed before the study. Instead, the power of the regression models was investigated afterwards using the software G*Power 3 [37]. The power of the test (1-β) for the regression models was >0.90 based on a medium effect size (f^2^=0.15), a significance level (α) of 0.05, 11 predictors and a sample size of 381 patients. Patients scheduled for general surgery declined participation or were missed being asked more often than those who participated in the study. This could possibly have had an effect on the results, especially since the general surgery patients were proportionally more classified in ASA class 2. A problem with prospective studies is that a number of patients drop out during the data collection. In this study, this might have been due to an age-related factor; it is possible that the younger patients experienced a more rapid recovery process and were back in their ordinary life, and therefore did not deem it important to complete the study assignment. The International Association of Ambulatory Surgery recommends follow-ups to be conducted up to about one month after the surgery [38]. Many patients have recovered by that time. More knowledge on recovery and HRQoL after day surgery is needed to estimate if this recommendation will be regarded as an optimal follow-up period. The proportion of men was larger among the non-responders. Possibly, some of them had agreed to participate before reflecting sufficiently on the commitment involved, and therefore decided to withdraw. Some patients did not fill out the questionnaires on all occasions, which resulted in their exclusion from the paired analysis; the varying sample sizes in the different analyses are due to this. However, on account of the large sample size no imputation of missing data was performed.

## Conclusion

In general, day surgery patients improve within two weeks. Orthopaedic day surgery patients are, however, at risk of experiencing a protracted postoperative period. Unfavourable recovery may have a negative impact on patients’ HRQoL, at least up to one month postoperatively. Regarding patients’ participation in the process of care, this knowledge may be important for health care professionals to consider.

## Competing interests

The authors declare that they have no competing interests.

## Authors’ contributions

KB, KK, MU and KÅ were responsible for the study design and the drafting of the manuscript. KB carried out the data collection and KB and KÅ analysed and interpreted statistical data. KB, KK, MU and KÅ have critically revised the paper and have given final approval of the version to be published. All authors read and approved the final manuscript.

## Pre-publication history

The pre-publication history for this paper can be accessed here:

http://www.biomedcentral.com/1472-6955/11/24/prepub
